# Generations of interdisciplinarity in bioinformatics

**DOI:** 10.1080/14636778.2016.1184965

**Published:** 2016-05-23

**Authors:** Andrew Bartlett, Jamie Lewis, Matthew L. Williams

**Affiliations:** ^a^ School of Social Sciences, Cardiff University, Glamorgan Building, King Edward VII, CardiffCF10 3WT, UK

**Keywords:** bioinformatics, interdisciplinarity, big data, scientific careers, collaboration

## Abstract

Bioinformatics, a specialism propelled into relevance by the Human Genome Project and the subsequent -omic turn in the life science, is an interdisciplinary field of research. Qualitative work on the disciplinary identities of bioinformaticians has revealed the tensions involved in work in this “borderland.” As part of our ongoing work on the emergence of bioinformatics, between 2010 and 2011, we conducted a survey of United Kingdom-based academic bioinformaticians. Building on insights drawn from our fieldwork over the past decade, we present results from this survey relevant to a discussion of disciplinary generation and stabilization. Not only is there evidence of an attitudinal divide between the different disciplinary cultures that make up bioinformatics, but there are distinctions between the forerunners, founders and the followers; as inter/disciplines mature, they face challenges that are both inter-disciplinary and inter-generational in nature.

## Introduction

The formation of scientific disciplines has been the subject of much sociological attention over the years (Lemaine 1976; Abir-Am 1985; Stichweh 1992; Lenoir 1997). A relatively recent trend has seen quantitative and qualitative transformations of some of these disciplines as they have become “big sciences,” and latterly “big data” sciences. de Solla Price (1963) coined the term “big science” to describe fundamental changes that occurred in the social organization of science in the second half of the twentieth century. Not only had science become a mass occupation, with scientific ideas penetrating our cultural and political systems, but in disciplines such as physics, by the mid-twentieth century, cutting-edge work in the field had changed from being “small” science – often conducted in one laboratory by a small team – to big science involving hugely expensive apparatus that required large teams and technical support (see Galison and Hevly 1992). More recently, biology has undergone an arguably comparable transformation in the wake of the Human Genome Project (HGP) (Bartlett 2008; Hilgartner 2013).^1^ At roughly the same time, in the second decade of the twenty-first century, we are now told that we are in the midst of an era of “big data” (see Borgman 2015), an era that will transform knowledge production practices across the academy, including even the social sciences (see Mayer-Schönberger and Cukier 2013; Hand and Hillyard 2014). Within this new big science, big data complex, a specialism emerged that can be thought of as emblematic of biology going big; the interdisciplinary research field of bioinformatics (Stevens 2011, 2013).^2^

While the historians of science, Strasser (2010), Suárez-Díaz (2010), and García-Sancho (2012), have traced the origins of “bioinformatics” to the 1970s, it was the impetus provided by the life sciences’ first modern “big science” project, the HGP, that prompted the rapid growth in bioinformatics and the acquisition of the first disciplinary “trappings:” journals, conferences, undergraduate courses, departments, and so on (Stevens 2013). The HGP provided funding, purpose – including moral cause (Bartlett 2008; Zwart 2008) – and brought together a diversity of scientists into an interdisciplinary nexus. Bioinformatics draws on the hinterlands of disciplinary knowledge (and culture) of not only biology and computer science, but also, among others, medicine, mathematics, and statistics. In the post-HGP era, an age of -omic sciences (such as proteomics and metabolomics) and “big data,” bioinformatics has acquired an epistemologically central role in the life sciences. While the wet lab produces *primary inscriptions*, transforming DNA, proteins, and so on into documentary traces (Latour and Woolgar 1986), it now does so on such a scale that bioinformaticians are needed to take this mass of traces and transform them into *secondary inscriptions* which can be biologically understood (Lewis and Bartlett 2013).

But, in what seems a paradox, with epistemic centrality has come a place on the institutional periphery (Lewis and Bartlett 2013). The new interdisciplinary researcher, working in bioinformatics, who perhaps calls herself a bioinformatician, finds challenges not just in the often unrecognized differences in knowledge and method, the mismatched salience (Collins 2001) between disciplines, but also of cultural differences (Lewis, Bartlett, and Atkinson, forthcoming). While much work on the problems of accomplishing interdisciplinarity has focused on epistemic differences and knowledge “deficits,” and while there has been some recognition of the cultural differences at play (see, for example, Reich and Reich 2006), there has been little discussion of the differences in value systems between “generations.”^3^ Different disciplinary cultures find value in different things, valuing different kinds of work (in the wet or dry lab, for example), outputs, and priorities (Penders, Horstman, and Vos 2008; Lewis, Bartlett, and Atkinson, forthcoming), but value systems also change between generations. Despite widespread valorization of interdisciplinarity as a “good” by agents of science policy (Lyall *et al.* 2011; Barry and Born 2013; Siedlok and Hibbert 2014; Callard and Fitzgerald 2016), the institutions of science have become accustomed to assessing scientific work on disciplinary lines (see, for example, the Research Excellence Framework in the United Kingdom). Against this background, bioinformatics is often seen as being neither good biology, nor good computer science, but, rather, as a service provider to biology (Lewis and Bartlett 2013). That bioinformaticians are reliant on the primary inscriptions produced by the biologists is one of the reasons why – along with existing institutional influence and the greater esteem in which biologists are held – in big data biology, power lies with the biologist (Lewis and Bartlett 2013; Leonelli 2016).

Despite this, interdisciplinarity is widely accepted as an increasingly important part of the post-HGP life sciences (Stevens 2013), and, indeed, is often regarded as a good in and of itself across the contemporary academy (Strathern 2006). Interdisciplinarity is regarded by some as a way to a more accountable science (Strathern 2004), and to more user-focused, innovative and economically productive ends (see the essays in Mirowski and Sent 2002). This is not to imagine that interdisciplinarity is in itself novel (Schaffer 2003, for example, describes instances from the history of science of interdisciplinarity in action), only to remark that its valorization at the level of science policy is a distinctive feature of the contemporary scene. In the case of the life sciences, the accomplishment of the HGP has been a catalyst for change, causing “traditional disciplinary boundaries to become blurred, or [to] break down, in the face of newly emerging sciences” (Diamond and Woodgate 2005, 239).

It has been argued that the accomplishment of the HGP has ushered in an era of “big science” in the life sciences (Collins, Morgan, and Patrinos 2003), with new techniques, technologies, and social organizations required for the collection of biological data in a routinized, large-scale manner. The story of the tensions, at varying scales, involved in building big biology projects has been rehearsed before (see Balmer 1993; Arribas-Ayllon and Bartlett 2014; Hilgartner 2013). Leonelli and Ankeny (2015), among others, have written of the short-term development of communities from interdisciplinary collaboration. This paper builds on these arguments by discussing the longer term temporality, or rather, perhaps, the “generationality” – in the sense of the biography of researchers – of the formation of bioinformatics, a consideration of which is essential for a science policy concerned with the crystallization of interdisciplines into disciplines themselves (see Jasanoff 2013 for a reflexive account of the formation of Science and Technology Studies (STS) as a/n inter/discipline). While this paper is not a reconstruction of the history of bioinformatics, but an exploration of generational differences within this interdiscipline, we must necessarily touch of the *story* told of the origin of this field. This is a story that for our purposes will, like the story of Tristram Shandy, begin with the coupling of its “parents.”

## Biographies and generations: marriage, birth, and childhood

Disciplines shape the structure of academia, mapping onto the institutional and educational structures by which academic life is organized and reproduced (Weingart and Stehr 2000; Whitley 2000). Interdisciplinarity^4^ arises – or perhaps *ought* to arise – when a problem or task falls in “between” the borders of these well-established disciplinary forms (Moran 2010). Calvert (2010) has identified two different forms of interdisciplinarity: “individual” interdisciplinarity, in which each scientist masters the knowledge and methodological domains of two or more disciplines, and “collaborative” interdisciplinarity (though we prefer the term collective interdisciplinarity^5^), which involves a group of scientists who pool their disciplinary expertises. Bioinformatics typically employs a mix of the two, with specialist bioinformaticians sitting in the “borderlands” between biology and computer science, working on projects alongside scientists from established disciplines.

The interdisciplinarity of bioinformatics has often been described as a “marriage” between biology and computer science (Cook-Deegan 1996), and quite often as a “shotgun marriage” at that. Marijuán (2002) wrote that “bioinformatics, computational biology, genomics, transcriptomics, proteomics, metabolomics, signalling science [ … were] the progeny derived from the shotgun marriage between molecular biology and computer science and engineering during the 1970s” (111). That is a lot of unplanned children, and quite some family tree. This form of interdisciplinarity is a top-down process, a result of conscious science policy, with those holding the shotgun sometimes the institutions and funding agencies, as Richard Jorgensen, quoted in *Nature*, recounts: “What the NSF has done is forced a kind of shotgun marriage between biologists and computer scientists” (Ledford 2009, 1048). However, it is often the case that the marriage is seen as resulting from the unescapable logic of the situation, with the shotgun being held by history, circumstance, and the nature of the problems presented by biology. Indeed, as Chow-White and García-Sancho (2012) have pointed out, the founding generation of genomics experienced this marriage as a “convergence,” as an organic, rather than synthetic, coming together of disciplines.

Not all births are straightforward, much less childhood and adolescence. Knorr Cetina (1999) has written of the “birth drama” of disciplines and of shared origin stories, while Franklin and McNeil (1993) have called such narratives “procreation stories.” Such a narrative is more complicated when that birth involves multiple partners (see Shrum, Genuth, and Chompalov 2007; Vermeulen 2010). Disciplines have a “biography,” and bioinformatics is no different. Ouzounis (2012) describes three phases of bioinformatics development inspired by the development of “big biology:” “Infancy” (1996–2001), “Adolescence” (2002–2006), and “Adulthood” (2007–2011).^6^ Just as with the biographies of people, when we tell the “life history” of a discipline, we also tell a story of the formation of self-identity, or *identities*. Elsewhere, we have discussed the tensions inherent in the coming-of-age of bioinformatics (Lewis and Bartlett 2013; Lewis, Bartlett, and Atkinson, forthcoming), though, like many others, we have tended to neglect the process of growth and development. This paper adds to our qualitative analysis by providing a snapshot of a discipline under development, unfolding, and “growing up” just less than a decade after the completion of the HGP. The paper draws on a survey of UK bioinformatics, and builds on a decade of ethnographic insight and interviews conducted while doing fieldwork with researchers working in and around UK academic bioinformatics.

That bioinformatics is a field that is still not fully mature is something noted by our survey respondents. The survey included a final “open text” question asking respondents to describe the “challenges” facing UK bioinformatics. One respondent used their answer to frame the question of maturation in terms of the uncertainty on the part of those outside the field as to the identity and character of the still-growing, “adolescent” field of bioinformatics:

[Bioinformatics is a] relatively young discipline, [it] may take a while before importance is recognised. There is confusion about what bioinformaticians can provide compared to statisticians/epidemiologists. (Senior Lecturer in Mathematics/Statistics, survey respondent)

As with any child of parents from different cultures – and while in bioinformatics’ case, the parents are many and varied, the principal parental roles are taken by biology and computer science – bioinformatics also has to find its identity *vis-à-vis* the cultures of its parents. Other aspects of our work on bioinformatics (Lewis, Bartlett, and Atkinson, forthcoming) note that cultural differences between these parental cultures have not always been taken into account. Different disciplinary cultures have different value systems^7^ (see Knorr Cetina 1999; Traweek 1992 for classic treatments of the role of culture in science). Furthermore, one of the recurring questions in our qualitative investigations of the field is that of “just what is bioinformatics?” This question often takes the form of: Is bioinformatics a discipline in its own right, or is it a service providing specialist technical support to biology and biologists? Unsurprisingly, the answer in the case of bioinformatics is most likely “a bit of both,” just as the range of roles (and careers) in the parent disciplines ranges from technician to professor through everything in between as well as other roles outside that spectrum. One of the survey respondents points this out:

[M]ost biologists who want to ‘do’ some bioinformatics (e.g. analyse array data) don’t have the time to learn the skills themselves without extensive training, and also find it difficult to collaborate with academic bioinformaticians due to their own commitments. There are huge gaps for essentially bioinformatics ‘technician’ to help out with multiple groups – partly funded by each group, or centrally funded. (Core Facility Manager in Bioinformatics, survey respondent)

It is the balance that is important. There *is* a need for “service” or “support” bioinformatics, with many universities establishing centralized infrastructure to provide this service. The tension in bioinformatics arises from a frustration of encultured expectations – that the education, training, expertise, and contributions of bioinformaticians would afford them the status and symbolic rewards of the research scientist (Lewis and Bartlett 2013). The frustration of these expectations by the demands for service bioinformatics produces an effect similar to that seen in technicians by Keefe and Potosky (1997).^8^

Of course, for all the usefulness of the metaphor of biography, a collectivity such as a scientific discipline differs from an individual in many ways. Important for our analysis is the simple fact that, over time, the constituent parts of a collective – the population of individuals – change in an identifiable way. As much as the history of bioinformatics can be *imagined* as the maturation of an individual, thinking sociologically, we are, in fact, talking about the development of a *culture*. And when we consider the development of a disciplinary culture, one of the clear lines that can be drawn is that between *forerunners*, *founders*, and *followers* ^9^ (after Ben-David and Collins 1966).

The distinction between forerunners, founders, and followers can be found in the “native” analysis of actors (the bioinformaticians who were our research participants) as well as that of sociological analysts. Consider this respondent’s reply when asked to consider the challenges to bioinformatics:

I wouldn’t say that there are any bioinformatics-specific challenges. As in any new area you have to invest a lot of energy to turn the area into a credible science in the eyes of outsiders who fund the whole thing. This can only be done by very special people. Having paved the road, these very special people leave and ‘just’ special people come. They do their job in [terms of] simplifying and explaining and introducing, and then they leave. Then the subject crystallizes enough to make-up a formal university curriculum. You get a larger and larger influx of ordinary people who work from the model provided by others, and [an] outflux of special people who made it happen.

Remember when ‘computer programming’ has suddenly stopped to mean anything? It was when they started to teach it in universities. Now we have the same situation in the field of bioinformatics. Higher quantity implies lower quality, it is mathematical law. So the challenge remains the same, but you get new names for it with every generation. (Bioinformatics Software Developer in Computer Science, survey respondent)

The professionalization of bioinformatics has, according to this “actor’s analysis,” inexorably led to a reduction in quality. The stabilization (and routinization) of a new field is described in terms of changes in the virtuosity (or mediocrity) of its practitioners. Developing a disciplinary identity and the institutional trappings^10^ of a conventional discipline are not, in this account, to be read as necessarily good things. The respondent suggests that this is something akin to a “universal law” in the development of disciplines. We, however, offer an alternative take, noting that while the professionalization and formalization of a field obviously change the make-up of the field as people follow the forerunners and founder, the presence of a reward structure and a bedrock of established practice can also work to attract quality people (even if they are “followers”), and offers the foundations for (at the very least) incrementally better work.

Regardless of pessimistic interpretations, the respondent does identify a generational difference within bioinformatics. On that much we agree. When analyzing the results of our survey of UK bioinformaticians, we split our sample according to when respondents had completed their undergraduate degree. We adopted a periodization relative to the HGP, taken *a priori* as a historically important period in the development of bioinformatics. This periodization is intended to reflect the differences in the life science milieu *vis-à-vis* the use of computers in biology.^11^ Respondents were grouped according to whether they gained their degrees before (prior to 1990), during (1990–2001), or after (2002 and later) the period of the HGP. Before the HGP, bioinformatics was a niche field of activity, during the HGP it developed rapidly, growing in importance and developing new tools and capabilities, and after the HGP, in the era of [gen]omic sciences, bioinformatics has become increasingly central – at least epistemologically central – to the practice of the life sciences. These changes provide us with generations that can be roughly described as consisting of forerunners, founders, and followers of the field (Ben-David and Collins 1966).

But, as has been shown (Lewis and Bartlett 2013), these forerunners, founders, and followers are not the product of a single culture. In our survey, we collected responses from people working in bioinformatics who had their scientific hinterland in a variety of disciplinary backgrounds. This has allowed us to look quantitatively at attitudes toward disciplinary formation based on culture – in this case, whether the respondent has arrived at bioinformatics from the life sciences or computer science – as well as generation, all of which complement our qualitative work (Lewis and Bartlett 2013; Lewis, Bartlett, and Atkinson, forthcoming). Our work points to the fact that the cultural differences at play in interdisciplinary work are not only those of different disciplinary cultures, but also “generational” differences. The nexus is thus inter-*generational* as well as inter-*disciplinary*.

## Methods

The primary data used in this analysis were derived from an online survey conducted during 2010 and 2011 of researchers working, in some way, in the field of bioinformatics. The Bristol Online Survey tool was used to design and distribute the questionnaire via email. Approximately 1000 scientists at UK research-intensive institutions were invited to complete the survey, each having been identified as involved in research, teaching, or administration of bioinformatics, with 326 completing the questionnaire. The survey was intended to explore the disciplinary “tensions” identified in our previous work, with 38 questions divided into 5 sections: Background,^12^ Training and Education, The Discipline of Bioinformatics, Credit and Reward, and Collaboration. The survey questions were designed in collaboration with “gatekeepers” working in bioinformatics.

Online surveys can rapidly generate results for exploratory research and they are now well established in the social sciences (Fielding, Lee, and Black 2008). We took advice from bioinformaticians who had participated in our qualitative research on both questionnaire design and sampling. Given the complexities in profiling the field of bioinformatics, our sampling frame was difficult to define. As a result, we adopted nonprobability sampling to derive the sample of respondents, using the self-selecting sampling technique. While sample bias is a fundamental shortcoming of nonprobability sampling, Meyer and Wilson (2009) note that this is often the only option available to researchers embarking on exploratory research. Furthermore, as the hypotheses tested in this analysis are concerned more with the existence of inter-variable relations and strengths of association than estimating population prevalence, the use of nonprobability sampling does not fundamentally weaken the design of the study (Dorofeev and Grant 2006). Moreover, our study is principally concerned with “soft” measures (attitudes, perceptions, opinions), which have no absolute validity (i.e. they cannot be compared with any authoritative external measure). However, Meyer and Wilson (2009) caution that sampling bias can still affect hypothesis testing if a sample is significantly uncharacteristic of the target population. Selective targeting was employed during survey recruitment to mitigate this potential problem. The research was conducted in line with the ethical guidance established by the Association of Internet Researchers. We made efforts to establish informed consent via the introduction page to the online survey. The research aims and objectives were clearly expressed and all respondents were informed that the data produced would be anonymized and would remain confidential.

The survey data built upon and were supplemented by qualitative interviews with biologists and bioinformaticians working in genomics conducted by Bartlett and Lewis across a number of projects over the past 10 years.^13^ This fieldwork, through close interaction with those working in data-intensive biological research, also allowed us to gain a degree of ethnographic insight into the practices, concerns, and values of the communities involved. In total, across these projects, we have conducted over 100 interviews with biological researchers working in large-scale biology – not all of which were directly relevant to the writing of this paper – as well as collecting fieldnotes and innumerable and unquantifiable “headnotes.”^14^

## Disciplinary identity of the sample

Of the 326 UK-based^15^ academics who completed the survey, 309 answered the question asking which discipline best describes the focus of their home department or research center. Of those responses, 20.7% (64) stated Bioinformatics; 31.4% (97) put Biology; 8.7% (27) Medicine; 12.3% (38) Computer Science; 3.9% (12) Mathematics; 7.8% (24) Statistics; and 15.2% (47) selected “Other.”

“Other” encompasses the handful of respondents who put Chemistry as the focus of their home department or research center, as well as Engineering and Artificial Intelligence. Thus, only a fifth of respondents were employed in a workplace the focus of which they would best describe as “bioinformatics.” As a relatively new field, this is hardly surprising. It does, however, support the idea of bioinformatics as a field that crosses disciplinary boundaries. With this in mind, survey participants in the study were asked which discipline(s) best described their work, rather than their workplace. The difference is worth noting.

Of the respondents who answered the question, 190 (approximately 60%) stated that Bioinformatics best described the discipline in which they worked. As we were alert to the heterogeneity of the discipline – and of individual [inter-]disciplinary identity within bioinformatics – rather than forcing respondents to choose a single category, the survey allowed respondents to select multiple categories.

Of course, all respondents in the sample were identified as working in bioinformatics in some form or other, given that they were included in the cohort of UK academic scientists who were invited to take part in the survey. What is notable about these figures is the number of respondents who declare that bioinformatics is one of the disciplines that *best* describes their work while working in a department/institute/research center, and so on that does not have bioinformatics as its main focus. A great amount of bioinformatics work is, necessarily and entirely predictably, done within other disciplinary settings. Our sample captures this phenomenon, though this is a phenomenon that is not unique to bioinformatics. This can be seen in our sample, as several of the other disciplines involved in bioinformatics – mathematics and statistics, for example – also appear to have a significant “diaspora,” at least considered in terms of our sample (Table 1).^16^

**Table 1.** Disciplinary identity.

	… best describes the focus of their *workplace*?		… best describes the focus of their *work*?		
Which discipline …	*n*	%	*n*	%^a^	%^b^
Bioinformatics	64	20.7	190	61.5	32.8
Biology	97	31.4	124	40.1	21.4
Medicine	27	8.7	30	9.7	5.2
Computer Science	38	12.3	73	23.6	12.6
Mathematics	12	3.9	28	9.1	4.8
Statistics	24	7.8	67	21.7	11.6
Other	47	15.2	67	21.7	11.6

Notes: The percentages listed in the “focus of workplace” columns are the proportions of respondents who selected each option. There are two percentages – %^a^ and %^b^ – listed in the “focus of work” column. %^a^ is the proportion of *respondents* who claimed that discipline as one of the foci of their work, while %^b^ is the proportion of total *responses* for each discipline.

In terms of the focus of our respondents' work, while 190 respondents selected Bioinformatics, the more established and stable categories of Biology, Computer Science and Statistics were, in combination, selected 264 times (some respondents, of course, ticked more than one box). The difference between working, place of work, and self-identification captures some of the tensions between disciplinary origins and interdisciplinary destinations in research careers (Delamont, Atkinson, and Parry 2000). For many of the more senior survey respondents and interview participants, bioinformatics was not their original discipline of study – necessarily so in the case of the forerunners and founders as cannot ride ready-laid disciplinary rails. The details of the following interview extract (collected as part of our wider work on the development of bioinformatics) may be idiosyncratic, but the general narrative is not atypical of the disciplinary history of someone “arriving” at bioinformatics from the biological sciences.

I was originally a zoologist, [and] became a microbiologist. Then, in the last three tears, I have no moved into bioinformatics. It has been a curious route, but basically it has been a conscious decision on my part to move into bioinformatics when I decided that [it] seems to be a productive area and it clearly has a lot of future. (Professor and Research Scientist in Biomedicine, interview participant)

Delamont, Atkinson, and Parry (2000) have discussed the relative stability of disciplinary identities. Their studies of the academic socialization of doctoral students in a variety of fields – in the sciences and social sciences – display the extent to which students and their supervisors working in interdisciplinary fields retain a strong sense of identification with their “home” discipline. While they may describe themselves *as working* in a field such as environmental sciences, they describe themselves *as being* a geologist, a hydrologist, or a chemist. In Artificial Intelligence departments, for example, people were likely to describe themselves as “really” mathematicians, engineers, or computer scientists. Of course, the relative “purity” or “stability” of the originating discipline is itself a matter of definition; it is not an inherent property of the field.

When asked to consider the statement that “Bioinformatics is a distinct discipline,” respondents were divided, with just under half claiming that they “Agree” (42.2%) or “Totally Agree” (5.4%). A smaller proportion “Disagree” (22.5%) or “Totally Disagree” (4.8%), but the fact that only about half of our respondents agree with the statement, and just under a third disagree provides us with clear evidence that, at the time that this survey was conducted, the field was still struggling to find a solid identity even among its own active participants, presenting us with an interesting snapshot of the field in the early post-HGP era.

When asked to consider the statement that “Bioinformatics is a service,” just under half (47.6%) “Agreed” or “Totally Agreed” with the statement, while 27.3% “Disagreed” or “Totally Disagreed,” with the remainder neither agreeing nor disagreeing. Agreeing with this statement does not imply approval for such status, as previous work (Lewis and Bartlett 2013) has shown. “Is” is not an “ought;” bioinformaticians can offer a “pessimistic” definition of bioinformatics while attempting to resist being written into the margins. Indeed, in response to the open-ended question in the survey asking respondents to consider the “challenges” faced by bioinformatics, several respondents bemoaned the positioning of bioinformatics as a service and what they felt was a lack of recognition of bioinformatics as a distinct specialism. Considering the perceived marginalization of bioinformatics as a service (to be used by biologists), we should remind ourselves of what Ben-David and Collins (1966) had to say:

the uninterrupted growth of a scientific field depends upon the existence of a scientific community permanently devoting itself to the field. Therefore a new idea is not sufficient to start the take off into sustained growth in a new field; a new role must be created as well. (451)

If the role is akin to that of a technician, the work to which bionformaticians will be devoted will be that of those who call upon their services. Some survey respondents suggest that a clear distinction between the service role and “disciplinary” work should develop:

Bioinf[ormatics] is primarily a tool and skill set that biologists should be able to apply and understand, while development and pushing boundaries of bioinf[ormatics] is what dedicated bioinformaticians do. For a broad and adequate provision of the Life Sciences in the UK with bioinf[ormatics] services, it is my view that ‘service’ bioinformaticians are required that can help PIs carry out their research using cutting edge methods, as there is a serious bottleneck in low availability of skilled bioinformaticians. (Lecturer in Biology, survey respondent)

That there is a diversity of views on the disciplinarity of bioinformatics, and the make-up of any such disciplinarity, is quite clear. The question is, what factors are associated with these very different views of bioinformatics? Are there inter-generational differences?

## Multivariate results

Ordered logistic regression analysis was conducted to determine associations between the predictor variables (see Table 2) and dependent variables of perceiving bioinformatics as a *discipline* (Model 1) and as a *service* (Model 2) (Table 3).^17^

**Table 2.** Descriptive statistics (*N* = 326).

		Sample	
Independent variables	Coding	*n*	%
*Controls*			
Gender	0 = Female	62	19.6
	1 = Male	254	80.4
Seniority	9 = Professor	79	24.9
	8 = Reader	21	6.6
	7 = Senior Lecturer	36	11.4
	6 = Lecturer	35	11.0
	5 = Research Fellow	37	11.7
	4 = Research Associate	34	10.7
	3 = Research Assistant	10	3.2
	2 = Postdoc Researcher	30	9.5
	1 = PhD student	35	11.0
*UG degree period*			
Pre-HGP	1 = Yes	102	39.7
During HGP	1 = Yes	122	47.5
Post-HGP	1 = Yes	33	12.8
*Discipline*^a^			
Bioinformatics	1 = Yes	195	59.8
Biology	1 = Yes	138	42.3
Medicine	1 = Yes	31	9.5
Computer Science	1 = Yes	81	24.8
Mathematics	1 = Yes	31	9.5
Statistics	1 = Yes	67	20.6
*Funding source*			
RCUK	1 = Yes	220	67.4
Charity	1 = Yes	138	42.3
NHS	1 = Yes	15	4.6
Commercial	1 = Yes	61	18.7
EU	1 = Yes	108	33.1

Note: Valid percentages reported.

^a^Respondents could select more than one discipline to describe their work.

**Table 3.** Ordered regression predicting perceptions of bioinformatics as a discipline and a service.

	Model 1: Discipline				Model 2: Service			
	*B*	SE	Wald	Exp(*B*)	*B*	SE	Wald	Exp(*B*)
**Dependent variable**								
Totally disagree	−1.13	.77	2.15		−2.47	.77	10.39	
Disagree	0.08	.76	0.01		−1.28	.76	2.83	
Neither agree nor disagree	0.76	.76	0.99		−0.67	.76	0.77	
Agree	2.64	.78	11.37		0.99	.76	1.69	
Ref: totally agree								
**Independent variables**								
*Controls*								
Gender	0.00	.19	0.00	1.0	0.09	.19	0.24	1.1
Seniority	−0.10***	.04	6.11	0.9	−0.06*	.04	2.62	0.9
*UG degree period*								
During HGP	0.01	.17	0.01	1.0	−0.29**	.18	2.77	0.7
Post-HGP	0.34	.31	1.18	1.4	−0.44*	.31	1.99	0.6
Ref: Pre-HGP								
*Discipline*								
Bioinformatics	0.07	.17	0.16	1.1	−0.50***	.18	8.01	0.6
Biology	−0.45***	.15	8.54	0.6	0.05	.15	0.09	1.0
Medicine	−0.01	.26	0.00	1.0	−0.10	.26	0.16	0.9
Computer Science	−0.18	.18	1.01	0.8	0.01	.18	0.00	1.0
Mathematics	−0.05	.25	0.05	0.9	0.41*	.25	2.66	1.5
Statistics	−0.26*	.19	1.99	0.8	−0.08	.19	0.17	0.9
*Funding source*								
RCUK	−0.32**	.17	3.69	0.7	−0.34**	.17	4.12	0.7
Charity	0.09	.16	0.33	1.1	−0.33**	.16	3.99	0.7
NHS	−0.07	.34	0.04	0.9	−0.26	.36	0.52	0.8
Commercial	0.16	.19	0.73	1.2	0.20	.19	1.06	1.2
EU	0.03	.16	0.03	1.0	−0.25*	.16	2.46	0.8
*Esteem indicators*								
Software	0.12**	.06	3.81	1.1	0.09*	.06	1.94	1.1
Funding	0.16**	.07	4.95	1.2	−0.11*	.07	2.30	0.9
Teaching	0.22***	.07	9.93	1.2	0.03	.07	0.13	1.0
Papers	−0.10	.09	1.26	0.9	−0.16**	.09	2.90	0.9
Service	−0.06	.06	0.90	0.9	0.01	.06	0.01	1.0
PhD supervision	−0.03	.08	0.10	1.0	−0.13*	.08	2.45	0.9
Conference	0.07	.08	0.88	1.1	0.04	.08	0.23	1.0
Patents	0.00	.09	0.00	1.0	0.09	.09	0.99	1.1
Commercial	0.03	.08	0.11	1.0	0.05	.08	0.37	1.1
*Modes of learning*								
Informal	−0.01	.11	0.00	1.0	−0.23**	.11	4.71	0.8
Formal	0.02	.07	0.14	1.0	0.03	.06	0.21	1.0
*Perceptions*								
Imp. bckgrnd medicine	0.03	.09	0.10	1.0	0.48***	.09	26.36	1.6
Imp. bckgrnd comp sci	0.12*	.09	1.85	1.1	0.09	.09	1.06	1.1
**Model fit**								
−2 log likelihood	598.890				615.910			
Model *χ*^2^	57.381				82.566			
df	28				28			
sig.	.001				.000			
*N*^a^	245				242			
Nagelkerke Pseudo *R*^2^		.22				.31		

Notes: *B* = coefficient (mean change in the response variable for one unit of change in the predictor variable while holding other predictors in the model constant); SE = standard error (the standard error around the coefficient for the constant); Wald = Wald Test; Exp(*B*) = the exponentiation of the *B* coefficient, which is an odds ratio (this value is given by default because odds ratios can be easier to interpret than the coefficient, which is in log-odds units).

^a^Reduction in sample size due to listwise deletion of cases necessary for regression requirements.

*Level of statistical significance: *p* < .10.

**Level of statistical significance: *p* < .05.

***Level of statistical significance: *p* < .01.

### Perceptions of bioinformatics as a discipline and a service

*Seniority*. Level of seniority emerged as significantly associated with perceiving bioinformatics as a discipline, with those survey participants in less senior positions being more likely to hold this perception. In other words, those holding relatively junior posts – those who are more likely to be followers, in academic and chronological terms – were more likely to consider bioinformatics to be a distinct discipline. Their encounters with bioinformatics are more likely to have been encounters with a field that has accumulated some of the institutional and cultural trappings of a discipline as part of the professionalization of courses.

*Undergraduate degree period*. Undergraduate degree period emerged as significant in relation to perceiving bioinformatics as a service. Respondents who received their degree during the HGP (1990–2001) period were significantly less likely to perceive bioinformatics as a service as compared to respondents who received their degree as in the pre-HGP (prior to 1990) period. Those classified as “during HGP” were nearly twice as likely to report this perception. It seems, therefore, that members of the forerunner generation were more likely to see bioinformatics as a service. This could be because, trained as they were before the coherence of bioinformatics as a distinct field, bioinformatics was just one more tool in the biologist’s toolbox, and a specialist in the use of only one particular tool is a service provider. By contrast, those of the founder generation – those who received their education during the HGP – were socialized into a life science milieu in which bioinformatics was in the process of becoming something distinct, not merely an adjunct to a biologist’s main work but the focus of work in and of itself.

*Discipline*. Respondents who identified biology as their field of study were significantly less likely to report perceiving bioinformatics as a discipline, while those who identified bioinformatics as their field of study were significantly less likely to perceive it as a service. In both cases, respondents were near twice as likely to hold these perceptions as compared to respondents in all other fields. The differences in “disciplinary” perception are not just generational, but cultural (Lewis, Bartlett, and Atkinson, forthcoming). The “biologists” are, understandably, more likely to see bioinformatics as a service, as an area of work that can be effectively black-boxed. Bioinformatics is just one more technique in their toolbox, and those who specialize in that technique – bioinformaticians – often occupy, at least in the biologists’ eyes, the role of technicians. On the other hand, the “bioinformaticians” in our sample – again, perhaps naturally enough – identified bioinformatics as having the status of a discipline. This provides further, broader evidence of the interdisciplinary conflict over status and symbolic reward in bioinformatics.

*Esteem indicators*. Those who considered software, funding, and teaching as indicators of esteem for their work were significantly more likely to perceive bioinformatics as a discipline. Those who indicated papers as an indicator of esteem were less likely to perceive it as a service. Though this is a confusing set of results, this is an indicator of the wider cultural conflict with the interdisciplinarity of bioinformatics. The production of software, in particular, is an academic activity that is valorized and rewarded in bioinformatics (and computer science) but not as easy to incorporate into the symbolic reward systems of biology. The interdisciplinary nature of bioinformatics means that cultures with quite different (at least within the bounds of academia) value (and valuation) systems are working together, and have to reconcile these differences.

*Perceptions*. Those who held the perception that medicine or biology was the most important disciplinary background for bioinformaticians were near twice as likely to perceive bioinformatics as a service. Conversely, those who held the perception that mathematics or computer science was the most important disciplinary background for bioinformaticians were more likely to perceive bioinformatics as a discipline; however, this association only approached conventional levels of significance. A bioinformatics that is driven by the needs of biology and medicine is a bioinformatics that is a disciplinary adjunct, a supplier of services to the life sciences. By contrast, a bioinformatics driven by computer science or mathematics is one that might have a greater appreciation of the value in the kind of academic outputs that a bioinformatician can produce independently of acting as a “technician,” providing specialist support to biologist. Developing new software, algorithms, statistical techniques – those with a background in computer science and mathematics will be better equipped by their disciplinary socialization to accord value to these kind of outputs.

## Qualitative findings: reconciling cultural differences

Powell *et al.* (2007) describe the ways in which fields of study acquire the “conventional insignia of a discipline;” among other things, names *do* matter. Of “molecular biology,” they write, “In part it was the multidisciplinary character of their activities, however, that provided its practitioners with a sense of disciplinary identity and a basis for constituting themselves along disciplinary lines” (12). It is not clear if that kind of “maturation” process has yet taken place in bioinformatics, but we do see a cohort of followers developing – those entering academic life after 2003 – who see bioinformatics differently from the forerunners and founders. The multi-/interdisciplinary nature of bioinformatics presents unresolved cultural challenges, which we have previously explored qualitatively (Lewis and Bartlett 2013). However, the analysis of the survey data, in this paper, highlights the way in which the kinds of academic activities that are accorded symbolic value differs depending on what respondents thought was the most important disciplinary background for bioinformaticians. One of the survey respondents suggests, with some pessimism, that the solution is closer physical integration:

Appropriate interactions between biology/medical groups and theoretical groups – often they are physically separated and also separated by boundaries through language/knowledge/culture. Co-location seems like an ideal way to overcome this challenge – but still does not always work – since groups often still stay in their safety zone. (Lecturer in Computer Science, survey respondent)

The cultural problems of interdisciplinarity are evident in other fields. Reyes-Galindo (2014) has noted that there is a significant social distance between experimentalists, data analysts, and theoreticians in “big physics,” even in avowedly collaborative projects. In the case of “big physics” – which has had much longer to get “big science” right (Galison and Hevly 1992) – the difficulties inherent in this cultural separation are resolved by the use of “ambassadors;” who undertake a “linguistic apprenticeship” in the culture of the other group. Similar suggestions are raised from respondents in our project to bridge differences between biologists and bioinformaticians.

You could almost see it as a consultancy role in that respect … Maybe there is a market for that, I don’t know. It needs a degree of management to show what you need to do … I think at the moment because you have not got so many people in this area, you end up having to do lots of roles in one person; the doing, the understanding and then the discussion and convincing … Personally, I like the idea of advising people what to do and making suggestions to them rather than sitting there nine to five with all the data sets. I do like the idea of educating people in bioinformatics. (PhD Student Studying Bioinformatics and Mathematical Biology, interview participant)

However, while in physics theoreticians often make claims to “virtual empiricism,” the charge that biologist sometimes level against bionformaticians is that bioinformaticians have next to no understanding of the biological significance of their findings, never mind the laboratory processes that produce the data. Do the mixing cultures in the borderland that is bioinformatics need ambassadors or consultants? Or is it enough to wait for a new hybrid culture, a creole (see Galison 1997), to develop? At least one of our respondents was explicit on the matter, and the parallels with physics:

Many experimental biologist[s] have the view bioinformatics as a service subject for biology. I am quite annoyed by the attitude of some experimental biologists towards computational scientists. I think it would be healthier if the relationship were more like that between experimental physicists and theoretical physicists. (Senior Lecturer in Computational Biology, survey respondent)

Addressing broader issues, as described, interdisciplinarity (and multi- and trans-disciplinarity) is widely seen as a “good.” The UK research councils expressly promote interdisciplinary work. But why is interdisciplinarity a “good”? As well as the extra-scientific “goods” described earlier – such as increased accountability and greater economic rewards – Hampton and Parker (2011) argue that interdisciplinarity, and more broadly, scientific synthesis, is a counter to hyper-specialization in science, that provides a means for exploiting an (over?^18^) abundance of data, increases the likelihood of truly novel work, and allows for complex problems (for example, in social or environmental systems) to be rendered tractable. In short, interdisciplinarity serves as a shorthand for – indeed, it is treated as a heuristic measure of – the degree to which work is innovative and capable of coping with “complexity.”^19^ Bioinformatics is a thoroughly contemporary example of disciplinary formation, arriving as it does in an age of big[ger] science, “big data,” digitization, and interdisciplinarity. The example that bioinformatics sets will offer an enlightening vision of new disciplinary formation in an academy much different from that examined by the classic studies of the subject.

## Discussion: on inter-cultural and inter-generational issues in interdisciplinarity

Biologists hold the upper hand in the borderlands of bioinformatics for several reasons. One, biologists hold cultural power – they are widely seen as the legitimate interpreters of the biological world. Two, they hold institutional power – they are well embedded in, and have the backing of, well-established institutional structures in academia. Three, as already alluded to, science is the practice of producing inscriptions (Latour and Woolgar 1986). Biologists control the resources – not just the funding, etc. (indeed, that is an aspect of their institutional power), but also the production and collection of data, the *primary inscriptions* upon which big (data-driven) biology rests (Lewis and Bartlett 2013). Bioinformaticians, as much as they seek to tell a story about the natural world, rely on their access to these data to produce secondary inscriptions.

In the case of the inter-cultural differences within this borderland, the values of biologists are those that appear to matter. But as we know, and as we see in this paper, bioinformatics is in the process of maturing; of, metaphorically at least, growing-up. Just as parents sometimes struggle to understand the value system of their children (and vice versa) so this generational change is accompanied by differences in attitudes and values. “Followers,” to continue to use Ben-David and Collins’s (1966) phrase, see the world differently. Importantly, they see bioinformatics differently. While the founders might lament these different values as evidence of the “mediocrity” of the next generation,^20^ it is the positions taken by the followers, and the positions into which the followers are put, that will define the identity of bioinformatics. Notably, as this paper demonstrates, this generation is more likely to see bioinformatics as a discipline and to resist definitions of bioinformatics as a service.

There are some lessons here, for those inhabiting, and governing, other interdisciplinary “borderlands.” The problems of interdisciplinarity are not only those of deficit (in knowledge) and difference (in culture), but also that the culture of disciplinary borderlands is one that is unstable, developing, *maturing*. Disciplinary cultures are, of course, not fixed or immutable. While the patterns laid down by the first generation to inhabit these borderlands – the founders – might persist, they will be changed by the follower generation/s, who will come to see this borderland as increasingly disciplined, and as a homeland rather than a place to which they have migrated.

The example of bioinformatics provides lessons not just for interdisciplinarity in general, but for the kind of interdisciplinarity that is expected to become increasingly important in an era of “big data.” This paper and previous work (Lewis and Bartlett 2013, for example) point to the role of power in determining which group is able to lay claim to the rewards of participating in a big data science. Biologists are able to claim and hold the position of legitimate interpreters of big data in the life sciences as they have institutional and cultural power. Biologists control the departments, funding panels, review boards, and so on that govern big data in the life sciences.

What will be the cultural and institutional power dynamics in other iterations of interdisciplinary big data science?

As an example that is close to home, Smith (2014) has argued that sociologists will be required in order to interpret (and critique) the output of “social data science,” and the Economic and Social Research Council (ESRC) have provided significant funding for big data social science, often with social scientists leading the project. But it might be naive of sociologists to expect that the question of which group is granted the right to interpret the results for policy and publics will be a matter of mere philosophical empiricism. Holmwood (2010) has argued that sociology lacks the “internal disciplinary integrity” that other disciplines possess, which might well lead to a situation in which non-sociologists from other disciplines – harder, more coherent disciplines held in greater esteem by the wider culture, and especially policy-makers – claim the right to interpret social data (Uprichard 2013). Without wanting to push the lessons that we might learn from bioinformatics too far, an exploration of “social data science” would have to consider not only questions of knowledge deficits and cultural difference – it can be argued that sociologists tend to hold inferior cultural and institutional positions when compared to scientists from the “harder” sciences – but also the development of a generation of “followers.” This generation of researchers will be natives of the increasingly disciplined landscape of “social data science” rather than practitioners of individual interdisciplinarity. Just as with the case of bioinformatics, the development of this new disciplinary sociality will be a fascinating field of study for sociologists. Even if these new researchers are our replacements.

Any pessimism aside, all these big data sciences, and interdisciplinary research areas in general, will undergo their own maturation, their own generational changes, with successive (and overlapping) generations of forerunners, founders, and eventually followers. In this paper, we have shown that, in bioinformatics, we can start to see the differences between these generations. Collaboration is a constant flux of conflict and competition as well as cooperation (Atkinson, Batchelor, and Parsons 1998). These conflicts are not just inter-cultural, but are also inter-generational. New biologists and bioinformaticists are increasingly socialized into a disciplinary landscape indelibly shaped by big data, a very different landscape from that which was the intellectual pastures of their forebears.

## Notes

1. There are older examples of “biological big science”, especially when we consider large-scale projects in which labor is distributed in the field rather than concentrated in laboratories – such as the ecological research described by Aronova, Baker, and Oreskes (2010) and the collaborative marine biology discussed by Vermeulen (2013). Neither are the pressures of dealing with “big data” entirely novel, as pointed out by Müller-Wille and Charmantier (2012).

2. Of course, not all bioinformatics is associated with “big science” projects; bioinformaticians are attached to all kinds of biological research. Indeed, one of the promises of biology in the age of big data is that the fruits of big science – the vast stores of data that these projects continue to produce – will be freely accessible, allowing anyone who is able to conduct the statistical analysis that is necessary to extract knowledge from these data to contribute to biology. Nevertheless, it was the HGP and its informational legacy that provided the impetus to the formalization of bioinformatics in courses, university departments, and “big” institutions such as the European Bioinformatics Institute (EBI).

3. That different generations might hold different value systems is something to which sociologists and historians ought to be sensitive. If not as professional observers of social life, then at the very least from its use as a source of dramatic tension in so many films and novels that deal with adolescence.

4. Inter-disciplinarity, multi-disciplinarity, cross-disciplinarity, and trans-disciplinarity are often used as if they are interchangeable synonyms. Stember (1991) offered definitions of these terms that are useful to sociologists. In this paper, we are discussing the interdisciplinarity of bioinformatics (in which there is a real synthesis of disciplinary expertise) even if the broader biological projects within which bioinformaticians work are, perhaps, more accurately “multi-disciplinary” (in which experts in different disciplinary domains work together).

5. We prefer “collective” as we feel that “collaborative” implies a degree of equality, collegiality, and shared ownership that, while it reflects the ideal type of scientific work, is not necessarily the case. Collective interdisciplinarity could be (and in some cases is) achieved by rigid, hierarchical methods, with specific expertise bought into the project on short-term or casual contracts, with “ownership” of the science claimed by the employer (see Lewis and Bartlett 2013).

6. In an earlier paper, Ouzounis and Valencia (2003) point out that bioinformatics extends much further back than 1996. The theoretical foundations of bioinformatics began to be set down in the 1970s, they argue, with the field taking some kind of shape during the 1980s and 1990s before an “explosion” in the wake of the HGP. See also García-Sancho (2012).

7. Aside from the classic works cited, see also work by Albert, Paradis, and Kuper (2015), who have shown that when disciplines meet, the culture of one can dominate the other. In their study of social scientists doing interdisciplinary work in medical research, most found that the value system – what counted as “academic excellence” – of social science did not match that of medical research, but that the value system of medical research was, as we might expect, dominant. Any change was “uni-directional”. In addition, Osborne and Holland (2009) discuss the differences between disciplines with regard to explicit guidelines for assigning scientific “authorship”. These are real, practical problems of cultural difference.

8. Indeed, there has been significant discussion of the “invisibility” of technicians in histories and other accounts of the accomplishment of scientific discovery. See, for example, Stewart (2008), or, of course, Shapin (1989).

9. We use Ben-David and Collins’ terminology as it allows us to capture not only the sequential but overlapping nature of the “generationality” of bioinformatics, but also because it suggests something of the “sentimental” orientation toward bioinformatics as a field and as a discipline.

10. Lyall *et al.* (2011) write: “Some interdisciplinary fields clearly ‘congeal’ to the point that they are recognized as disciplines in their own right with a shared epistemological base and associated professional markers such as journals and learned societies” (19).

11. Bartlett, who completed an undergraduate degree in Biology in 1999, notes that his degree contained little in the way of familiarization with the use of computers for biology. His MSc in Human Genetics, awarded in 2002, was heavily weighted toward the use of bioinformatics tools. Of course, in this case, the differences are not only temporal, but also of increasingly advanced and specialized training.

12. The background section provided us with data on the demography, education, and employment of the cohort. Among other things, this allowed us to, as described, periodicize the sample, grouping the participants into rough “generations”.

13. Wyatt *et al.* (2016) propose greater conciliation between quantitative and qualitative research as ways of studying the sociology of biomedical science.

14. Jackson (1990) reported that many of the anthropologists that she interviewed do not see their fieldnotes as any kind of discrete and concrete “thing”; they were not the “data” in and of themselves. Rather, they see their written notes as being incomprehensible without the “headnotes” of the researcher who was there in the field, interacting with members of the community being studied. While we cannot claim to have achieved true “interactional expertise” (Collins 2004) in any post-genomic bioscience community, the role of headnotes in any ethnographic research cannot be ignored.

15. The survey was limited to UK-based scientists in order to minimize the possibility that national differences in academic cultures, institutional structures, funding arrangements, and so on would prove to be confounding variables.

16. By “diaspora”, we mean people with a particular disciplinary identity working in a setting with a different disciplinary focus. As we can see from Table 1, only in the cases of Biology, Medicine, and Computer Science are the majority of respondents whose work is best described by those disciplines working in departments with the same disciplinary focus. As this is not a survey designed to capture the distribution of these disciplines, these proportions should not be taken to represent the actual “diasporic” populations.

17. Results from correlational analyses (not shown), and tolerance statistics and variance inflation factors showed that there were no problems with multi-collinearity among the independent variables. All models met the assumption of parallel lines and model statistics (Pearson and Deviance) in all cases indicated a robust fit to the data. Table 3 presents the results of the models.

18. Biologists in the post-HGP era have written of “drowning” in data (for example, Roos 2001).

19. “Complexity” appears to have been taken by some as the defining characteristic in all manner of problems in the late-modern, post-Cold War epoch (see, for example, Urry 2005).

20. A view of subsequent generations that is, at least apocryphally, as old as the written word.
